# Supplementary material for: Identification of photoperiod-induced specific miRNAs in the adrenal glands of Sunite sheep (Ovis aries)
Source: Front Vet Sci. 2022 Jul 22;9:888207. doi: 10.3389/fvets.2022.888207 (PMC9354845; doi:10.3389/fvets.2022.888207)
Supplement: Supplementary file 1 [file Data_Sheet_1.ZIP › Supplementary file/miRDeep2 file.zip!/miRDeep2 file/Know_Structure/LP42A2/oar-mir-17.pdf]

miRBase precursor : oar-mir-17
Total read count : 4098
oar-miR-17-5p read count : 4028
remaining reads : 70

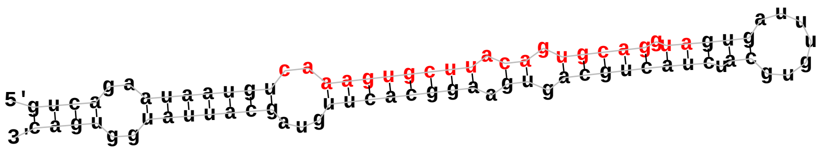

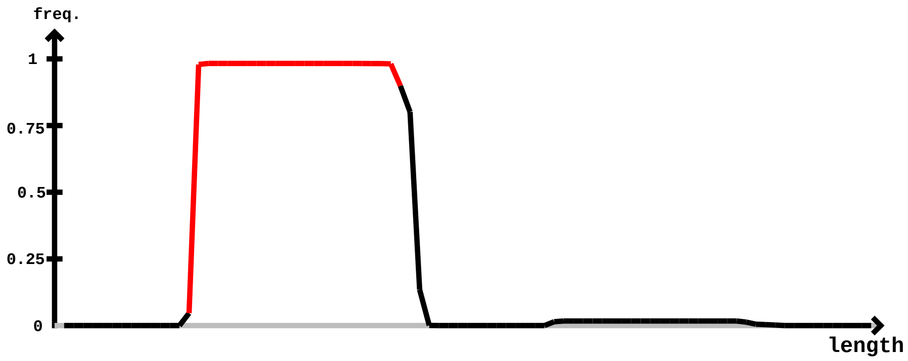

oar-miR-17-5p

5'- gucagaauaaugucaaagugcuuacagugcagguagugauuugugcaucuacugcagugaaggcacuuguagcauuauggugac -3'	exp		
((((..(((((((..((((((((.((.(((((.(((((.......)).)))))))).)).))))))))...)))))))..))))	reads	mm	sample
............ucaaagugcuuacagugcagg...	1	0	seq
............ucaaagugcuuacagugcaggu..	50	0	seq
............ucaaagugcuuacagugcaggua...	50	0	seq
............ucaaagugcuuacagugcagguag..	83	0	seq
............ucaaagugcuuacagugcagguagu...	7	0	seq
.............caaagugcuuacagugc..	1	0	seq
.............caaagugcuuacagugca...	1	0	seq
.............caaagugcuuacagugcagg...	4	0	seq
.............caaagugcuCacagugcaggu..	3	1	seq
.............caaagugcuuacagugcaggu..	286	0	seq
.............caaagugcuuacagugcaggua...	343	0	seq
.............caaagugcuCacagugcaggua...	2	1	seq
.............caaagugcuuacagugcagguag..	2620	0	seq
.............caaagugcuCacagugcagguag..	17	1	seq
.............caaagugcuuacagugcagguagu...	538	0	seq
.............caaagugcuCacagugcagguagu...	4	1	seq
.............caaagugcuuacagugcagguagug..	2	0	seq
..............aaagugcuuacagugcaggua...	4	0	seq
..............aaagugcuuaUagugcagguag..	5	1	seq
..............aaagugcuCacagugcagguag..	1	1	seq
..............aaagugcuuacagugcagguag..	5	0	seq
..............aaagugcuuacagugcagguagu...	1	0	seq
....acugcagugaaggcacuugu..............	17	0	seq
....acugcagugaaggcacuugua.............	32	0	seq
....acugcagugaaggcacuuguag............	6	0	seq
....acugcagugaaggcacuuguagc...........	1	0	seq
....acugcagugaaggcacuuguagca..........	3	0	seq
....cugcagugaaggcacuuguagc...........	6	0	seq
....cugcagugaaggcacuuguagca..........	5	0	seq
